# Analytical performance assessment and improvement by means of the Failure mode and effect analysis (FMEA)

**DOI:** 10.11613/BM.2020.020703

**Published:** 2020-04-15

**Authors:** Leonor Guiñón, Anna Soler, Mónica Gisell Díaz, Rosa María Fernández, Nayra Rico, Josep Lluís Bedini, Aurea Mira, Luisa Alvarez

**Affiliations:** 1 Quality Department, Biomedical Diagnostic Center, Hospital Clínic of Barcelona, Barcelona, Spain; 2 Biochemistry and Molecular Genetics Department, Biomedical Diagnostic Center, Hospital Clínic of Barcelona, Barcelona, Spain; 3 Direction, Biomedical Diagnostic Center, Hospital Clínic of Barcelona, Barcelona, Spain

**Keywords:** risk management, FMEA, sigma metric, analytical performance, quality control

## Abstract

**Introduction:**

Laboratories minimize risks through quality control but analytical errors still occur. Risk management can improve the quality of processes and increase patient safety. This study aims to use the failure mode and effect analysis (FMEA) to assess the analytical performance and measure the effectiveness of the risk mitigation actions implemented.

**Materials and methods:**

The measurands to be included in the study were selected based on the measurement errors obtained by participating in an External Quality Assessment (EQA) Scheme. These EQA results were used to perform an FMEA of the year 2017, providing a risk priority number that was converted into a Sigma value (σ^FMEA^). A root-cause analysis was done when σ^FMEA^ was lower than 3. Once the causes were determined, corrective measures were implemented. An FMEA of 2018 was carried out to verify the effectiveness of the actions taken.

**Results:**

The FMEA of 2017 showed that alkaline phosphatase (ALP) and sodium (Na) presented a σ^FMEA^ of less than 3. The FMEA of 2018 revealed that none of the measurands presented a σ^FMEA^ below 3 and that σ^FMEA^ for ALP and Na had increased.

**Conclusions:**

Failure mode and effect analysis is a useful tool to assess the analytical performance, solve problems and evaluate the effectiveness of the actions taken. Moreover, the proposed methodology allows to standardize the scoring of the scales, as well as the evaluation and prioritization of risks.

## Introduction

In the field of healthcare, the clinical laboratory is one of the areas that has made many efforts to minimize errors that may affect patient safety. Errors made in laboratories are approximately 0.3%, which is much lower than the percentage of errors in other areas of medicine (*1*). Despite this, taking into account the high number of laboratory tests that are usually carried out, this percentage of error can total millions of erroneous results *per* year. Although it is unlikely, some of these errors can lead to adverse effects on patients (*2*).

Errors can occur at any step in the testing process. However, in the last few years it has been found that most of the laboratory errors occur in the extra-analytical phases. This is because the vast majority of the strategies adopted to minimize risks have been focused on reducing failures in the analytical phase, such as the design of an internal quality control (IQC) plan or the participation in external quality assessment (EQA) schemes (*3*). Despite these strategies, nowadays the estimated percentage of analytical errors is approximately 23% of the total errors produced in laboratories (*4*). This indicates that it is necessary to improve analytical performance, therefore efforts to mitigate these errors should not cease.

A requirement of the international standard for accreditation of clinical laboratories, the International Organization for Standardization (ISO) 15189:2012, is the incorporation of risk management principles aimed at patient safety (*5*). The last version of the ISO 9001:2015 standard, frequently implemented in clinical laboratories, has also incorporated risk management to improve the quality of the processes (*6*). There are ISO directives and Clinical and Laboratory Standards Institute (CLSI) documents that provide guidance on risk management (*7*-*10*). Although these standards do not indicate which methodology should be used, the failure mode and effect analysis (FMEA) is the most applied (*2*).

Failure mode and effect analysis is a risk management tool for identifying possible failures that can occur and for solving known errors, analysing the causes and effects of the failures, and eliminating or reducing the most relevant ones by proposing control measures (*11*). After identifying and listing all the possible failure modes, the risk must be estimated using different factors. Clinical and Laboratory Standards Institute EP23A “Laboratory Quality Control Based on Risk Management” recommends a 2-factor risk model considering only the occurrence and the severity of a failure. However, to assess the analytical performance in medical laboratories a 3-factor risk model can be more appropriate (*12*). This model considers also the detectability, as the probability of not detecting a failure. Then, a value is assigned to each risk factor according to a numerical scale. Higher scores are assigned to those risks with a higher occurrence, higher severity and lower probability of detection. To identify the high priorities for action, the values assigned to the occurrence, severity and detectability are multiplied, thus obtaining an index score named risk priority number (RPN). Risk mitigation actions are more urgently needed for those risks with a higher RPN. After the implementation of these actions, the RPN must be calculated again to assess their effectiveness (*3*, *11*). Despite being the most used methodology, FMEA has shown some drawbacks such as the lack of standardization of scoring scales, subjectivity when assigning values to each risk factor or the lack of a unified criterion to assess the RPN value obtained (*13*).

It has been postulated that the Sigma metric, a tool that clinical laboratories have been using for some years to measure the effectiveness of its quality control procedures, could also be useful for the risk management of the analytical phase (*14*). One of the advantages of the Sigma metric is that it allows an objective assessment of the process performance and can also be used as a benchmark to compare the results obtained universally. The objective of the Six Sigma model is to reduce the variability of the processes, in such a way that six standard deviations can fit within the established tolerance limits of the process. Reducing variation, fewer defects (the results that fail to meet the specified tolerance limits) are generated so a performance improvement of the process is achieved. In order to use Sigma metric as a benchmarking scale, the number of defects produced must be expressed in defects *per* million opportunities (DPMO), and this must be converted into a Sigma value. The higher the Sigma value is, the lower the variation of the process. Six Sigma levels have been described (*15*). A Sigma value of 6, recognized as the world-class quality, corresponds to 3.4 DPMO. Sigma values between 5 and 6 are considered of excellent quality. Sigma levels of 4, 3 and 2 correspond to a good, moderate and poor quality, respectively. Sigma values below 2 indicate an unacceptable quality. It has been described that the number of defects in the analytical phase due to quality control failures (IQC and EQA) is around 3 to 4-Sigma (*15*). Outside of health care, a Sigma value of 3, which corresponds to 66,807 DPMO, has been described as the minimum acceptable quality for routine operation (*14*). Currently, as clinical laboratories perform a high number of tests, the same standards might be applied (*16*). Obtaining lower Sigma values would indicate the need to implement improvement actions.

The objective of this study was to carry out an FMEA of the analytical phase to assess the analytical performance of the measurement procedures, implement actions to mitigate the risks and evaluate their effectiveness.

## Materials and methods

### Materials

The study was performed in the Biomedical Diagnostic Center of the Hospital Clinic of Barcelona, Spain, in the period between 1st of January 2017 and 31st of December 2018. In the study were included all measurands whose measurement errors (ME) exceeded the allowable total error (TEa) in any of the surveys of the EQA scheme “Serum biochemistry” organized by the Spanish Society of Laboratory Medicine (SEQC). The following measurands were evaluated: alkaline phosphatase (ALP), direct bilirubin (DBIL), chloride (Cl), creatine kinase (CK), creatinine (CREA), high density lipoprotein (HDL), lactate dehydrogenase (LD), potassium (K), sodium (Na) and total protein (TP). The materials analysed were: i) quality control materials provided by the EQA scheme and ii) IQC materials: Liquid Assayed Multiqual level 2 and 3 (Bio-Rad Laboratories, Marnes-la-Coquette, France).

### Methods

All tests were run on ADVIA Chemistry XPT System analyser (Siemens Healthcare GmbH, Erlangen, Germany). From each EQA survey, the ME were obtained. It was calculated (Equation (Eq.) 1) as the distance in percentage from the value reported by the laboratory to the target value (instrument group mean):

ME (%) = [(Result – Target value)] x 100 / Target value

The selection of the allowable total error was preferably based on the biological variability (BV) (minimum, desirable or optimal values) (*17*). When the BV model was so demanding that the laboratory was not possible to achieve the minimum values, the criteria of the Clinical Laboratory Improvement Amendments (CLIA) was adopted (*18*). Table 1 shows the TEa set by the laboratory for the measurands included in this study.

**Table 1:** Total error quality specifications established by the laboratory

**Measurand**	**Source of Quality Specification**	**TEa (%)**
ALP	Desirable BV	12.04
DBIL	Optimal BV	22.30
Cl	CLIA	5.00
CK	Optimal BV	15.20
CREA	Desirable BV	8.87
HDL	Desirable BV	11.63
LD	Desirable BV	11.35
K	Minimum BV	8.40
Na	CLIA	2.79
TP	Minimum BV	5.40
BV – biological variability. CLIA – Clinical Laboratory Improvement Amendments. TEa – allowable total error. ALP – alkaline phosphatase. DBIL – direct bilirubin. Cl – chloride. CK – creatine kinase. CREA – creatinine. HDL – high density lipoprotein. LD – lactate dehydrogenase. K – potassium. Na – sodium. TP – total protein.		

Failure mode and effect analysis of the years 2017 and 2018 were performed using a 3-factor model. In order to assign objective values to the occurrence, severity and detectability, they were calculated using data from the EQA and the IQC, as follows:

1) Occurrence (O) was calculated (Eq. 2) as the percentage of errors (results whose ME exceeded the TEa) with respect to the number of surveys of the EQA scheme:

O (%) = Number of errors x 100 / Number of surveys

Those measurands that did not exceed the TEa in any of the EQA surveys were not included in the FMEA, since the occurrence value would have been zero.

2) Severity (S) was calculated (Eq. 3) as the difference expressed as a percentage between the ME and the TEa. A ME higher than TEa means a significant deviation that could lead to a false diagnosis or inadequate treatment, so the risk for the patient safety would rise as that difference increases.

S (%) = (ME – TEa) x 100 / TEa

When for a certain measurand there was more than one ME higher than the TEa, the average of ME was calculated.

3) Detectability (D) was calculated (Eq. 4) from the probability of error detection (Pde) provided by the Westgard Advisor module available in Unity Real Time v.2.0, a quality control data management software from Bio-Rad Laboratories (*19*). With this purpose, we obtained the Pde for both concentration levels of IQC and calculated the average. Since Pde is the probability of detecting an error, the detectability was calculated as the complementary value of the Pde (1 - Pde), thus obtaining the probability of not detecting an error. When the Pde value was 1, it was transformed into 0.999 in order to obtain data for the calculation. Results were expressed as a percentage.

D (%) = (1 – Pde) x 100

Following the methodology published by Sten Westgard, risk factors were expressed as a percentage to obtain the RPN on a scale of 1 to 1 million. Therefore, an estimation of the number of DPMO was obtained (*15*). Then, the DPMO was converted into a Sigma value (σ^FMEA^) to standardize the evaluation of the results and the prioritization of actions, providing a better assessment of the risks that are acceptable than the one provided by the RPN. The DPMO value (described in the Six Sigma conversion tables) closest to the DPMO obtained provides the σ^FMEA^ (*20*).

The laboratory investigated the possible causes of failure for those measurands with a σ^FMEA^ lower than 3. Thus, a root cause analysis by means of the repeated “whys” query tool was performed (*21*). After the investigation, some actions were taken to decrease the number of DPMO produced, such as applying an equation to correct systematic error or increasing the frequency of IQC concentration level 3 testing (3 times a day).

An FMEA of the year 2018 was performed to evaluate the effectiveness of these measures and to identify new residual risks. A σ^FMEA^ between 3 and 4-Sigma or higher would indicate the success of the corrective measures.

## Results

Tables 2 and 3 show the ME obtained from the EQA surveys in the years 2017 and 2018 respectively.

**Table 2:** Measurement errors expressed as a percentage obtained in the EQA surveys of the year 2017

	**ME (%)**											
**Measurand**	**Jan**	**Feb**	**Mar**	**Apr**	**May**	**Jun**	**Jul**	**Aug**	**Sep**	**Oct**	**Nov**	**Dec**
ALP	8.91	3.96	2.43	5.20	1.31	10.16	11.79	18.85	11.08	14.93	17.66	15.84
DBIL	14.60	30.47	24.02	1.44	4.49	4.63	4.02	0.39	5.81	0.85	7.29	0.23
Cl	1.08	3.39	1.21	2.17	1.54	0.26	1.96	0.70	0.63	2.10	1.75	7.81
CK	16.12	9.29	11.54	1.70	2.90	4.81	2.21	3.00	4.16	2.62	1.80	7.13
CREA	8.45	2.49	0.95	1.93	4.62	0.39	2.54	9.46	0.13	5.80	2.92	3.49
HDL	17.47	3.52	9.28	10.61	9.87	0.52	14.75	9.22	3.05	9.09	1.39	5.55
LD	4.79	6.03	5.39	6.15	7.33	3.68	5.52	4.37	5.49	3.95	5.42	12.61
K	1.86	1.98	0.73	3.23	0.74	1.19	1.15	0.21	1.01	0.21	1.56	11.62
Na	1.37	1.24	0.01	0.98	0.95	0.54	0.22	0.48	1.46	3.84	2.59	6.87
TP	0.35	2.34	0.97	1.38	0.65	7.07	0.31	4.65	1.84	2.93	1.00	1.78
ME – measurement error. ALP – alkaline phosphatase. DBIL – direct bilirubin. Cl – chloride. CK – creatine kinase. CREA – creatinine. HDL – high density lipoprotein. LD – lactate dehydrogenase. K – potassium. Na – sodium. TP – total protein.												

**Table 3:** Measurement errors expressed as a percentage obtained in the EQA surveys of the year 2018

	**ME (%)**											
**Measurand**	**Jan**	**Feb**	**Mar**	**Apr**	**May**	**Jun**	**Jul**	**Aug**	**Sep**	**Oct**	**Nov**	**Dec**
ALP	6.01	10.55	13.45	5.14	5.85	4.61	8.66	8.25	5.38	9.38	21.09	4.35
DBIL	2.09	10.02	4.57	0.59	2.61	1.92	6.37	0.26	2.59	3.89	16.66	10.74
Cl	2.71	4.00	1.17	2.62	0.64	5.82	0.07	1.16	0.45	0.61	0.51	0.28
CK	3.64	1.63	3.21	0.54	2.94	2.29	8.27	7.40	1.59	4.76	2.34	1.52
CREA	0.74	1.90	6.92	3.13	2.68	1.22	4.49	7.47	1.90	3.98	11.65	5.85
HDL	0.27	2.37	0.88	0.39	3.35	0.53	26.50	0.57	0.13	12.37	0.90	2.53
LD	4.12	22.50	2.95	0.61	1.44	1.19	1.95	1.24	0.01	0.69	2.26	1.34
K	0.81	4.40	0.06	4.48	2.73	4.19	0.49	0.71	0.25	1.92	0.23	2.65
Na	0.46	3.80	1.61	1.31	0.65	2.84	2.36	0.53	0.09	1.19	0.05	1.55
TP	3.85	2.54	0.47	3.29	3.45	2.87	1.48	0.47	1.81	1.57	2.16	3.52
ME – measurement error. ALP – alkaline phosphatase. DBIL – direct bilirubin. Cl – chloride. CK – creatine kinase. CREA – creatinine. HDL – high density lipoprotein. LD – lactate dehydrogenase. K – potassium. Na – sodium. TP – total protein.												

The results of the FMEA of the year 2017 are shown in Table 4. Ten measurands were evaluated. Alkaline phosphatase and Na presented a σ^FMEA^ of less than 3 and only CK was close to the world-class quality. The results of the FMEA of the year 2018 are shown in Table 5. Only six measurands were included. None of the measurands presented a σ^FMEA^ below 3. The results showed that σ^FMEA^ for ALP and Na increased.

**Table 4:** FMEA of the year 2017

**Measurand**	**Occurrence (%)**	**Severity (%)**	**Pde**	**Detectability (%)**	**DPMO**	**σ^FMEA^**
ALP	33.33	39.70	0.200	80.00	105,856	2.7
DBIL	16.67	22.17	0.948	5.25	1940	4.4
Cl	8.33	56.20	0.500	50.00	23,407	3.5
CK	8.33	6.05	0.999	0.10	5	5.9
CREA	8.33	6.65	0.610	39.05	2163	4.4
HDL	16.67	38.52	0.431	56.90	36,537	3.3
LD	8.33	11.10	0.200	80.00	7397	3.9
K	8.33	38.33	0.904	9.65	3081	4.2
Na	16.67	91.94	0.274	72.65	111,346	2.7
TP	8.33	30.93	0.426	57.45	14,802	3.7
Pde – probability of error detection. DPMO – defects *per* million opportunities. σ^FMEA^ – Sigma value. ALP – alkaline phosphatase. DBIL – direct bilirubin. Cl – chloride. CK – creatine kinase. CREA – creatinine. HDL – high density lipoprotein. LD – lactate dehydrogenase. K – potassium. Na – sodium. TP – total protein.						

**Table 5:** FMEA of the year 2018

**Measurand**	**Occurrence (%)**	**Severity (%)**	**Pde**	**Detectability (%)**	**DPMO**	**σ^FMEA^**
ALP	16.67	43.44	0.569	43.15	31,247	3.4
Cl	8.33	16.40	0.625	37.50	5122	4.1
CREA	8.33	31.34	0.595	40.50	10,607	3.8
HDL	8.33	67.11	0.449	55.10	30,802	3.4
LD	8.33	98.24	0.999	0.10	82	5.3
Na	16.67	19.00	0.582	41.80	13,239	3.7
Pde – probability of error detection. DPMO – defects *per* million opportunities. σ^FMEA^ – Sigma value. ALP – alkaline phosphatase. Cl – chloride. CREA – creatinine. HDL – high density lipoprotein. LD – lactate dehydrogenase. Na – sodium.						

## Discussion

In the present study, a 3-factor risk model was used to perform the FMEA. The proposed methodology allowed us to assess the analytical performance of the laboratory methodologies, detecting poor analytical performance of ALP and Na, which led us to implement improvement actions. The actions taken in order to reduce the number of DPMO were different for the two measurands according to the results of the root-cause analysis. Therefore, for the bias on the EQA results of ALP from August until December 2017, that was due to a lot-to-lot reagent variation, the action taken by the laboratory was to apply an equation to correct the systematic deviation until the reagent lot changed. The elimination of the bias led to a higher probability of detection in 2018 (from 0.200 to 0.569) and a reduction of the occurrence of errors in the EQA surveys. In the case of Na, a poor performance of IQC concentration level 3 was observed. The action taken was to increase the frequency of IQC concentration level 3 testing. The modification of the quality control procedure in 2018 increased the probability of error detection (from 0.274 to 0.582) and the errors committed in the EQA surveys had a lower severity. Because of the actions carried out in 2017, σ^FMEA^ obtained for these measurands in the FMEA of the year 2018 were between 3 and 4-Sigma, indicating an improvement of the analytical performance.

Other authors have applied the FMEA methodology to assess the analytical performance of clinical chemistry measurands (*22*-*25*). As a conclusion, all of them stated the usefulness of the risk assessment to detect if it was necessary to adjust the quality control procedures in order to improve the analytical performance. However, due to the lack of standardization of the FMEA methodology, it is not feasible to compare the results of the present study with those obtained in their studies. They also used a modified FMEA and the Sigma metric in order to overcome the drawbacks of the FMEA. However, their methodology was neither entirely objective nor easy to standardize mainly because the value assigned to the severity was based on personal experience (*22*-*24*). Furthermore, those studies do not explain the risk mitigation actions proposed nor even verify the effectiveness of actions implemented. Only Capunzo *et al.*, that performed an FMEA of the analytical process of three measurands (glucose, total cholesterol, and total bilirubin), listed the improvement actions performed and reassessed the risks once these actions were applied (*25*). As in the present study, they achieved a risk reduction (measured as RPN) for all measurands after implementing actions such as increasing the IQC frequency analysis or changing the procedure of preparation, storage and use of the calibrators.

It is important to remark that all the factors used to perform the FMEA depend on the TEa established by the laboratory. Therefore, in order to analyse correctly the results obtained, the laboratory must also assess whether the established tolerance limits are the right ones. Depending on the TEa established by the laboratory, it is possible to move from a σ^FMEA^ of less than 3 to a σ^FMEA^ of more than 3 or *vice versa*, as it occurs in other studies in which they have assessed the importance of selecting a proper TEa to obtain a reliable Sigma value that represents analytical performance (*26*). As it has been shown in other studies the current technology does not always allow to reach quality specifications based on BV, even though these specifications should be the objective (*27*). Therefore, to assess the results correctly, it should be considered whether the goals set by the laboratory are realistic or not. The evaluation of the analytical performance of the measurement procedures may not be adequate if the quality specifications established by the laboratory are too demanding. According to the authors, laboratory quality departments and responsible laboratory personnel should set together adequate goals.

The proposed methodology allowed us to identify those magnitudes with a less satisfactory analytical performance and, once the causes were detected and the improvement actions implemented, verify their effectiveness. Performing an FMEA using EQA and IQC data allows to standardize how the scoring scales are made. This means a reduction in the time needed by the staff to calculate the risk score and ensures the treatment of risks under the same criteria. Furthermore, this study shows that the Sigma metric can be used in the FMEA to standardize the prioritization of risks, as well as to measure the change in risk score after having made risk mitigation actions.
